# Creativity research in medicine and nursing: A scoping review

**DOI:** 10.1371/journal.pone.0317209

**Published:** 2025-01-08

**Authors:** Alex Thabane, Sarah Saleh, Sushmitha Pallapothu, Tyler McKechnie, Phillip Staibano, Jason W. Busse, Goran Calic, Ranil Sonnadara, Sameer Parpia, Mohit Bhandari

**Affiliations:** 1 Department of Health Research, Evidence, and Impact, McMaster University, Hamilton, Canada; 2 Department of Surgery, McMaster University Medical Center, Hamilton, ON, Canada; 3 Department of Anesthesia, McMaster University Medical Center, Hamilton, ON, Canada; 4 DeGroote School of Business, McMaster University, Hamilton, ON, Canada; 5 Department of Surgery, University of Toronto, Toronto, ON, Canada; 6 Vector Institute for Artificial Intelligence, Toronto, ON, Canada; University of Verona, ITALY

## Abstract

**Background:**

Creativity fuels societal progress and innovation, particularly in the field of medicine. The scientific study of creativity in medicine is critical to understanding how creativity contributes to medical practice, processes, and outcomes. An appraisal of the current scientific literature on the topic, and its gaps, will expand our understanding of how creativity and medicine interact, and guide future research.

**Objectives:**

We aimed to assess the quantity, trends, distribution, and methodological features of the peer-reviewed on creativity in medicine.

**Methods:**

We searched the MEDLINE, EMBASE, and PsycINFO databases for peer-reviewed primary research publications on creativity in medicine. Screening, full-text review, and data extraction were performed independently and in duplicate by pairs of reviewers, with discrepancies resolved by a third reviewer. We performed descriptive analyses, graphically displaying the data using charts and maps where appropriate.

**Results:**

Eighty-one studies were eligible for review, enrolling a total of 18,221 physicians, nurses and midwifes across all studies. Most research on creativity in medicine was published in the last decade, predominately in the field of nursing (75%). Researchers from Taiwan (22%) and the United States (21%) produced the most eligible publications, and the majority research was cross-sectional in nature (54%). There was substantial variability in the definitions of creativity adopted, and most studies failed to specify a definition of creativity. Forty-five different measurement tools were used to assess creativity, the most popular being divergent thinking tests such as the Torrance Test of Creative Thinking (24%) and Guilford Creativity Tests (16%).

**Conclusions:**

Peer-reviewed scientific research on creativity in medicine, mostly conducted in the nursing profession, is sparse and performed on variable methodological grounds. Further scientific research on the topic, as well as the development of medicine-specific definitions and measurement tools, is required to uncover the utility of creativity in the medical domain.

## Introduction

Creativity fuels societal progress and innovation. Just consider the significance of Sir Alexander Fleming’s discovery of penicillin, or Paul Lauterbur’s work on magnetic resonance imaging. For these reasons there has always been an interest in creative individuals and the qualities that they possess; yet our understanding of creativity has only recently become an interest of the scientific community.

JP Guilford’s 1949 address to the American Psychological Association (APA) can be considered as the official starting point of modern creativity research [1]. At the time, less than 0.002% of titles in the PsycINFO database, were related to creativity [1]–a reflection of just how little we knew about, and were interested in, the scientific study of creativity. Since then, researchers have learned much about what creativity is, its processes, and the qualities associated with it. The two-criterion conceptualization of creativity, consisting of novelty and usefulness, is now widely accepted as the ‘Standard Definition’ of creativity through which we can assess the creativity of ideas, products, and solutions [2]. Theoretical models of creativity such as the Componential Theory of Creativity [3], the Investment Theory of Creativity [4], and the Amusement Park Theory of Creativity [5], have also enhanced our understanding of the processes and antecedents leading to such creative products. There has also been substantial research demonstrating the association between intelligence, personality, motivation, and environmental factors on creativity, which give us a better idea of what qualities makes people creative [6–10]. As our general understanding of creativity has grown and its importance defined, many domains, such as education, engineering, and business, have begun to prioritize creativity [11–14]. However, one field that has yet to fully embrace creativity is the field of medicine.

Medicine is the science of diagnosis, prevention, alleviation, and cure of human illness. And where human life is involved, safety takes precedence. Creativity, in contrast, involves risk and uncertainty [15, 16]. It follows that creativity in medicine (CIM) may be unethical, especially when it fails to generate positive outcomes. Yet, the field of medicine is increasingly meeting complex challenges requiring creative solutions. The COVID-19 pandemic is just one example of the value of CIM: medical professionals, businesses, and society at large relied on creativity to manage transmission, attend to the sick, and develop life-saving interventions for a novel disease. For instance, continuous positive airway pressure devices were successfully–and creatively–repurposed to provide respiratory support for COVID-19 patients where ventilators were in short supply. From an innovation standpoint, CIM has led to several transformational advancements in patient care, from the advent of organ transplantation to the hormonal treatment of prostatic cancer [17]. Creativity can be useful in medicine; many physicians have already acknowledged this [18]. However, if we are to cultivate CIM, more scientific research is needed to understand its best use-cases, how to validly measure it, and how to cultivate it in medical professionals.

An understanding of the existing evidence on CIM, including the identification of areas of interest, current gaps in knowledge, and limitations in the existing approaches to the study of CIM, is necessary to guide future research. To meet this need, we aimed to conduct a scoping review of the scientific literature, identifying the quantity and types of evidence, temporal and geographical trends in CIM research, and the methodological characteristics of CIM research.

## Methodology

We conducted this scoping review in accordance with the JBI guidance for scoping reviews [19], and reported the review in accordance with the Preferred Reporting Items for Systematic reviews and Meta-Analyses extension for Scoping Reviews (PRISMA-ScR) [20].

### Identifying the research questions

We set out to answer the following research questions:

1. What is the quantity of peer-reviewed primary research on CIM?
2. How has research productivity in CIM progressed over time, by number of articles published per year?
3. Where is CIM research being conducted, by country?
4. What types of study designs are used in CIM research?
5. What is the distribution of CIM research across sub-domains of medicine (e.g., medical education, nursing, surgery)?
6. What methodologies are being used in CIM research, including definitions of creativity and measurement tools for creativity?

### Search strategy

We performed an electronic database search of the MEDLINE, EMBASE and PsycInfo databases from inception to September 27, 2023. A combination of keywords and Medical Subject Heading (MeSH) terms relating to creativity, medicine, and medical personnel were used to design the search strategies, tailored to the index structure of each respective database (**S1 Appendix**). Additionally, we searched the references of eligible articles for additional publications, and used the Connected Papers platform to identify similar papers. The Connected Papers platform calculates a similarity metric based on overlapping citations and references to cluster similar papers together [21].

### Eligibility criteria

Included studies were primary, original research articles with the objective of studying or measuring creativity in the context of medicine, that were published in a peer-reviewed journal. In this study, we limited our scope to the medical context in the population of physicians, nurses and midwifes. To guide study eligibility, we defined creativity as the “interaction between aptitude, process, and environment by which an individual or group produces a perceptible product that is both novel and useful as defined with in a social context” [22]. However, given the variable definitions of creativity in the literature [23] and our objective of exploring the definitions of creativity used in CIM research, we included studies that deviated from this definition, but explicitly aimed to study creativity as part of the outcomes or objectives of the study.

The following exclusion criteria were applied:

1. Studies of ‘creative interventions’ (i.e., creative art, creative writing, dance).
2. News articles, interviews, opinion papers, workshops, presentations, posters, dissertations, editorials, conference abstracts, books, or book chapters.
3. Studies not published in English.

### Study selection

The screening of titles and abstracts, and subsequent full-text review of potentially eligible studies, were performed independently and in duplicate by a pair of reviewers (AT, SS, SP). We resolved discrepancies by discussion to achieve consensus, with intervention of a third reviewer as necessary. Interrater agreement between reviewers during full-text review was substantial (κ = 0.743).

### Data charting

We performed data charting independently and in duplicate using a standardized extraction form created on the Covidence platform. We resolved any discrepancies during the data charting process by discussion to reach consensus, with discrepancies resolved by the lead author. Extracted data included: surname of the 1^st^ author; title of the article; name of the journal; year of publication; country in which the study was conducted; medical specialty; study objective; study design; definition of creativity used; description of the population; total number of participants; creativity measurement tool used.

### Collating, summarizing, and reporting the results

We summarized the results of the literature search, screening process, and full-text review using a PRISMA diagram [24]. We performed a narrative synthesis of the data, representing the data in the form of charts and maps where appropriate (using Microsoft Excel). Where applicable, continuous data is represented as means with standard deviation (SD), or median with interquartile ranges (IQR) when non-normally distributed; categorical data is presented as counts (percentages).

## Results

### Search results

**Fig 1:**
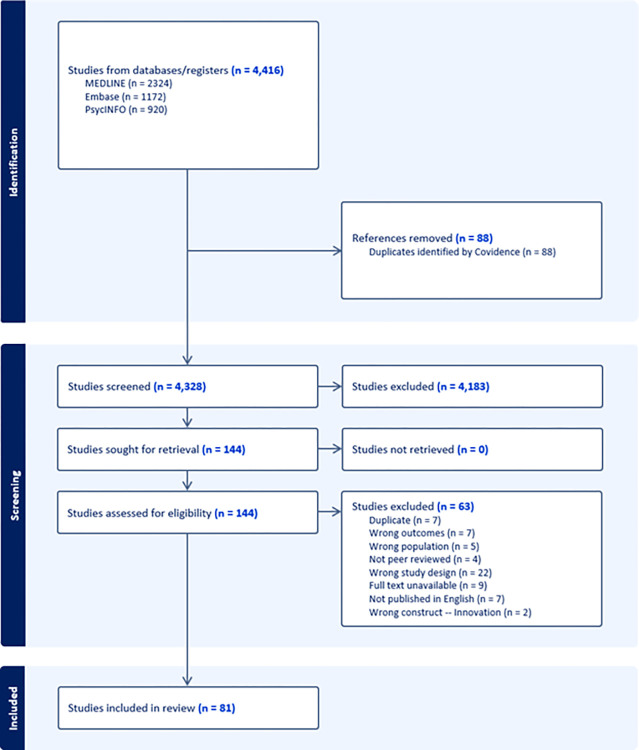
PRISMA diagram of the study selection process.

After the removal of 88 duplicates, 4,328 unique citations were included for screening. After screening and full-text review, we included 81 primary research studies on CIM, that enrolled a total of 19,352 participants (**Fig 1**). Seven studies were excluded due to not being published in English, and 22 due to not being primary research articles. A comprehensive list of the included and excluded studies can be found in **S1 File**.

### Temporal and geographic trends in research output

**Fig 2:**
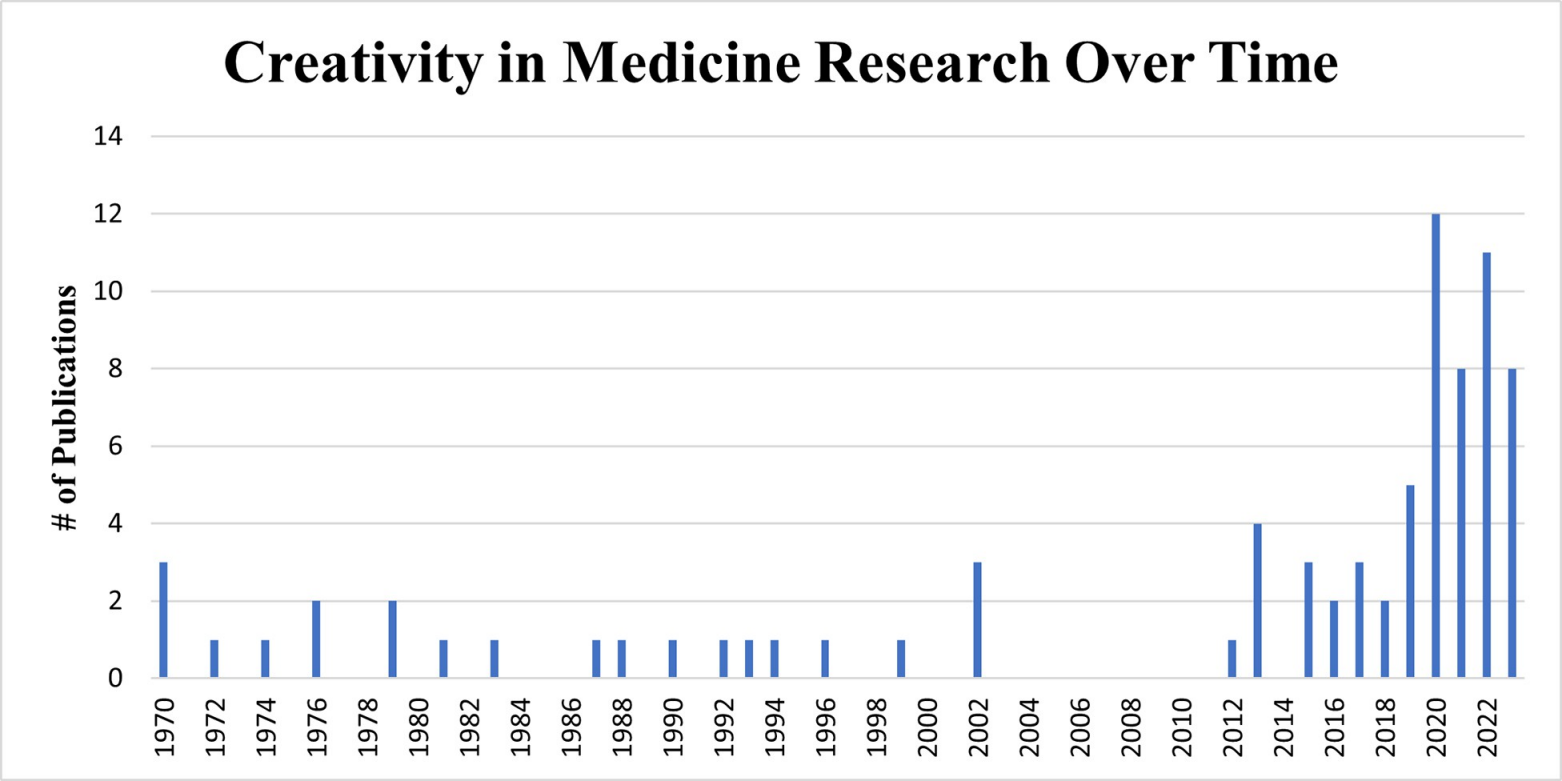
Progression of research productivity over time.

The first peer-reviewed creativity research articles in the medical field were published in the 1970s [25–29]. For the first 30 years, research efforts were sparse and limited: only 22 studies were published from 1970 to 2002. Between 2003 and 2011, no primary research on CIM was published. However, a noticeable increase in research productivity has occurred in the last decade: since 2012, 59 studies (73% of the total) have been published. **Fig 2** illustrates the trend in CIM research productivity over time.

Despite global participation in CIM research, an asymmetry exists in research productivity by country: the current literature is dominated by publications from Taiwan (n = 18; 22%) and the United States (n = 17; 21%). Iran (n = 8; 10%) and China (n = 7; 9%) have contributed to the evidence base to a lesser extent. The other 21 countries involved produced between 1 and 4 peer-reviewed publications in total. A world map of the geographic distribution of creativity research publications in the field of medicine can be found in **Table 1**.

**Table 1:** Geographic distribution of creativity research in medicine.

Country	# of Publications
Taiwan	18
USA	17
Iran	8
China	7
South Korea	4
Spain	4
Canada	2
Pakistan	2
Sweden	2
Turkey	2
Australia	1
Egypt	1
Hong Kong	1
India	1
Italy	1
Japan	1
Lebanon	1
Netherlands	1
Norway	1
Romania	1
Saudi Arabia	1
South Africa	1
Thailand	1
Tunisia	1
United Kingdom	1

### Study designs

**Fig 3:**
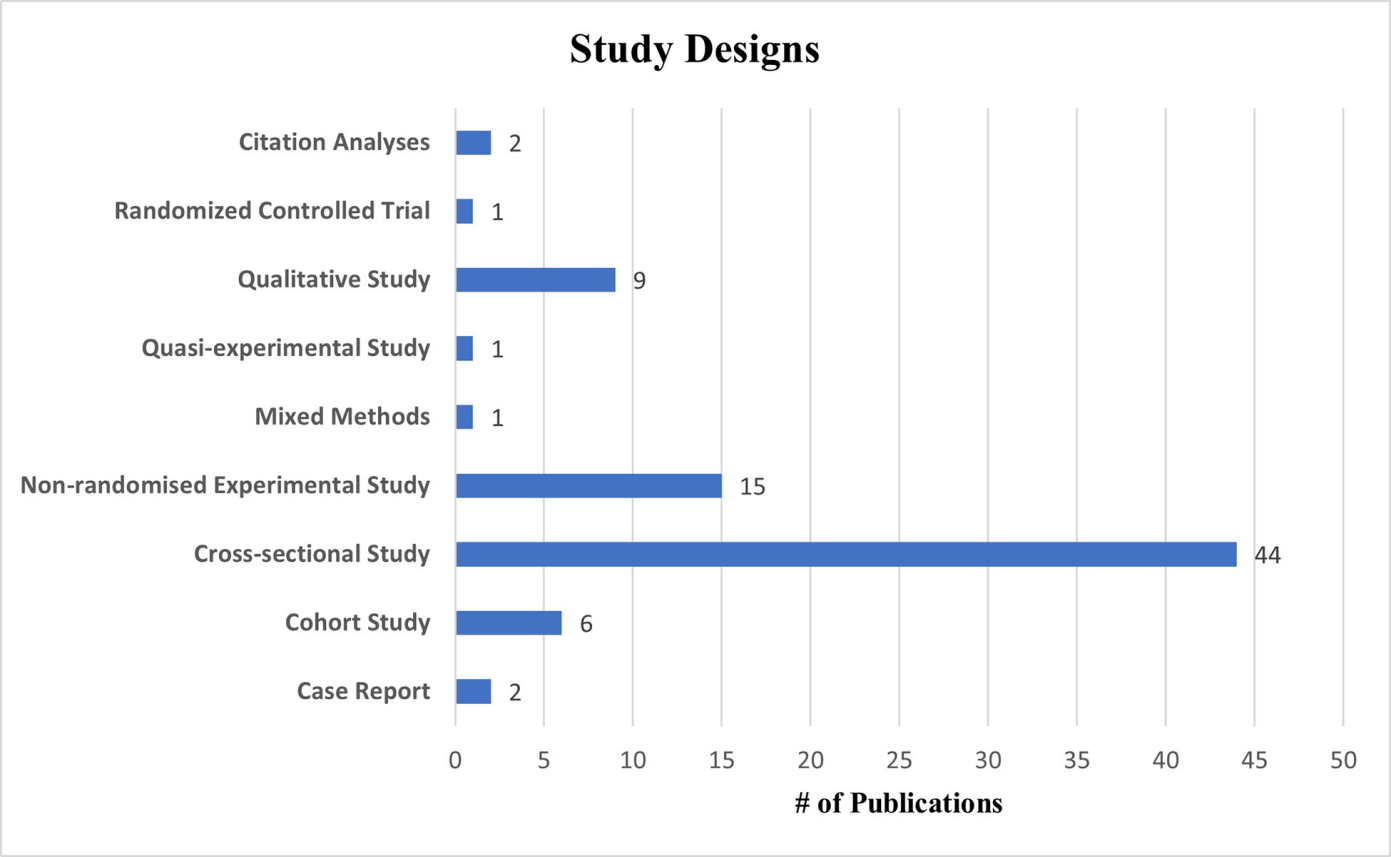
Study designs used in creativity in medicine research.

The cross-sectional study design, in which all data is collected at a single point in time, is the most widely used study design in CIM research (n = 44; 54%). Non-randomized experimental studies were the 2^nd^ most common design utilized (n = 15, 19%), some of which involved the implementation of novel teaching curriculums to improve creativity [25, 30–32]. Qualitative study designs (n = 9; 11%) and cohort studies (n = 6; 7%) were utilized to a lesser extent. Very few case reports (n = 2), citation analyses (n = 2), randomized controlled trials (n = 1), quasi-experimental studies (n = 1) and mixed-methods studies (n = 1) were found. A summary of the study designs used in CIM research is illustrated in **Fig 3**.

### Nursing

**Fig 4:**
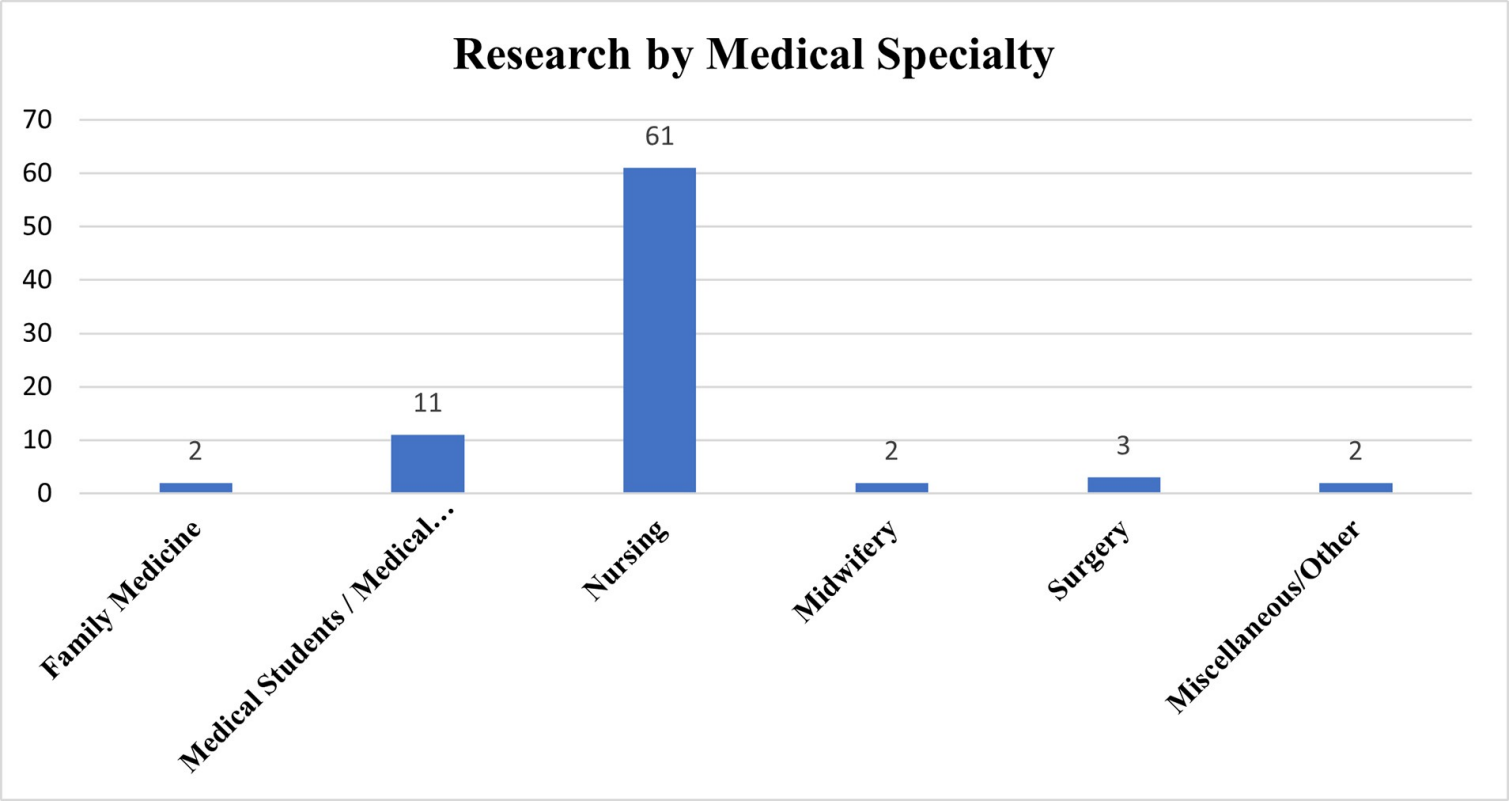
Creativity research output by medical specialty.

CIM research has been dominated by the nursing domain, which contributes more than 75% of the existing literature (n = 61). **Fig 4** illustrates the distribution of creativity research in medicine, by specialty.

The work of Taiwanese researchers, particularly in the last 5 years, is responsible for most of the creativity research in nursing. One author, Hsing-Yuan Liu, is responsible for one-fifth of the existing peer-reviewed CIM research–all published within the last 5 years (n = 16; 20%). The works of Liu span a range of areas, from creativity pedagogy in faculty members [33–35], to individual student creativity [36–40], to nursing team creativity [39, 41–45]. Her body of work also includes the development of a novel assessment tool for team creativity in nursing students, which has demonstrated strong reliability [46].

### Medical education

Despite nursing being at the vanguard of CIM research, only 11 studies been published on creativity in medical education. Two studies in medical students–a 1976 study on creative potential [47] and 2002 study on creative thinking and academic achievement [48]–constituted the entire body of creativity research in medical education between 1970 and 2010. However, an uptick in research on creativity in medical education in the last decade is indicative growing interest in, and appreciation for, creativity in medical training. The value of creativity in adapting to the changing landscape in medicine and communicating with patients has been explored in both cross-sectional and qualitative work [18, 49]. Strategies for cultivating creativity have also been investigated [50–52]. Assessments of trait creativity and divergent thinking suggest a desire to understand the creative potential of medical students [53, 54], perhaps in pursuit of increased innovation [55].

### Family medicine, surgery & other specialties

Research in other medical specialties is paltry. We found 3 studies in surgery [56–58], 2 studies in family medicine [28, 59], 2 studies in midwifery [58, 60], and 2 studies including a heterogeneous group of medical professionals [61, 62]. Areas of inquiry included burnout [58], quality of patient management [59], physician satisfaction [28], and research productivity [56, 57].

### Definitions of creativity

In the study of an abstract phenomenon, the definition the researcher adopts is influential with respect to measurement and the interpretation of findings. About (n = 40; 49.4%) of studies made explicit the definition of creativity they used. In those that did, there was substantial variation across studies. The idea of creativity being something novel and valuable/useful was commonly adopted [33, 36, 50, 60, 62–65], and is in keeping with current conceptualizations of creativity [2]. The definition of creativity as the ability to problem solve was also common [66–68]. We identified one domain-specific definition of the creative physician, who was characterized by the ability to view each patient and problem as unique, be comfortable with ambiguity, persistent in the exploration of verbal and non-verbal cues, and aware of patient emotions [28]. By and large, CIM research is conducted using variable definitions of creativity, and often lacking an explicit definition of creativity entirely.

### Measurement

Most studies (n = 64; 79%) aimed to assess creativity in some fashion. We found 45 different assessment tools and techniques used in the literature; tests of divergent thinking, an operational construct in JP Guilford’s Structure of Intellect (SOI) model of intelligence [69], dominates the creativity measurement landscape in medicine. The Torrance Test of Creative Thinking (TTCT), a divergent thinking test and the most popular assessment tool of creativity, was used the most (n = 11; 14%). Guilford creativity assessments (n = 7; 9%), also measuring divergent thinking, were frequently used. Some studies performed assessments of creative affect and personality, via tools such as the Affective Components of Creativity Scale (n = 3). The Barron-Welsh Art Scale, a Freudian-based creativity assessment tool, was administered in 3 studies.

Where creativity was measured as a function of problem-solving, tools utilized included the Cassidy/Long Problem-Solving Scale (n = 2) and Gordon Creative Problem-Solving Test (n = 1). Expert ratings as measure of creativity were sparingly adopted (Consensual Assessment Technique, n = 1). Measures of creative self-efficacy, such as the Creative Self-Efficacy Scale (n = 2), were uncommon. For the measurement of team creativity, we found the Farh team creativity scale (n = 6; 7.4%) to be most administered. To assess the creative climate/environment, researchers utilized the Creative Climate Questionnaire (n = 2), the Creative Environment Perceptions Scale (n = 1), and Creative Team Climate Scale (n = 1).

## Discussion

We have performed the first scoping review of creativity research in the domain of medicine, exploring the quantity, trends, distribution, and methodological features of the existing peer-reviewed literature. 81 studies on the topic have been published, the majority in the domain of nursing, with most employing a cross-sectional design. A few peer-reviewed publications exist in medical education, and hardly any in other areas of medical specialization. The definitions of creativity and measurement tools used to conceptualize and assess creativity are highly variable, indicating a lack of homogeneity in the theoretical assumptions underpinning CIM research.

Relative to the total body of medical research [70, 71], CIM research is very limited: this suggests a lack of appreciation or consensus of the utility of CIM. Whilst physicians and nurses agree that creativity is an important skill required in medical professions [72, 73], more research exploring its clinical utility is required. We found several studies which studying the utility of creativity in improving diagnostic accuracy, adapting to changing environments, providing personalized care, and mitigating burnout [18, 58, 74]. Studies exploring the benefits of creativity from the patient perspective–in improving patient outcomes, for example–could further uncover its value.

Taiwanese and American researchers produced more than 20% of the total peer-reviewed CIM research each. This is likely due to funding and institutional support for research. The USA is a world leader in research and development (R&D) expenditure and scientific publication productivity [75, 76]. The research productivity of Taiwan, which has a much smaller economy and R&D expenditure, importantly illustrates the power of institutional support for stimulating CIM research. The Creative Education White Paper, released in 2003, mandated the Taiwanese government to focus on improving creativity in its students, which led to an influx of courses, interventions, and research into creativity [35, 77]. If CIM research is to grow, government and educational institutions, hospitals, and medical professionals all have a role to play in prioritizing and supporting it. Creating research grant opportunities could be a way to stimulate research on the topic, as the receipt of funding and grants can have a strong effect on improving publication output [78].

Creative potential is not static: it changes with age [79, 80], and can be trained [81, 82]. The cross-sectional design of most studies on creativity in medicine is therefore an important limitation of the current literature. Cross-sectional studies cannot capture the temporal fluidity of creativity, and limit the ability of researchers to make causal inferences [83]. Studies indicate a possible decline in creativity across medical training [26, 84]; if we are to understand the effects of medical training on creativity, longitudinal studies, in large populations, exploring the change in creativity from medical school through residency to professional practice is needed.

There is substantial heterogeneity in the definitions of creativity and measurement tools used. This complicates the interpretation of findings, as well as comparison between studies. The challenges of creativity measurement have long been acknowledged by psychologists [85]; to adequately measure creativity and creative potential in medical professionals, a operationalized definition specific to the domain of medicine, or specific medical specialties, would be helpful. This will likely require the use of existing definitions in the creativity literature combined with input from medical specialists to adapt existing definitions to the medical domain. Similarly, medicine-specific measurement tools should take into account the other aspects of creative potential specific to the medical domain, beyond the domain-general factors associated with creativity such as divergent thinking, intelligence, personality, and motivation identified in existing creativity theories and the empirical literature [4, 7–9].

Our study has several strengths. Firstly, we conducted a comprehensive search of 3 large databases and utilized the Connected Papers platform, in addition to perusing the references of relevant articles, to identify possible eligible articles. Secondly, we conducted all screening, full-text review, and data extraction independently and in duplicate, which has been found to reduce the number of missed studies and number of errors during data extraction [86, 87]. Thirdly, we assessed the trends and distribution of research, types of study designs used, and the definitions and measurement tools that were adopted. In doing so, we have identified the gaps in the current evidence base and limitations in methodological approaches, which will help guide future research efforts.

Like other reviews, our study has several limitations. Firstly, we excluded 7 articles that were not published in English. This was done for pragmatic reasons, as English was the primary language of the reviewers responsible for screening and data extraction. This may have introduced a language bias, particularly in our assessment of the geographical distribution of CIM research. However, the exclusion of non-English studies is justified, as including them would only provide a small increase in the number of studies while requiring significant resources (i.e., time; cost; translation expertise). Secondly, we excluded doctoral theses on CIM, such as Ann Hart’s work on nursing performance [88]. However, we chose to exclude as we sought to provide a scope of the peer-reviewed literature only, which is typically higher quality and thus more credible.

## Conclusion

Creativity is valuable in medicine, yet we still know relatively little about it from a scientific research perspective. The peer-reviewed literature on CIM is sparce, with only 81 published studies, mostly in the field of nursing. Taiwanese and American researchers have produced over 40% of the existing research, largely driven by the efforts of one researcher. There is substantial, and problematic, heterogeneity in the definitions of creativity and measurement tools used in CIM research. More longitudinal studies are required, and domain-specific definitions and measurement tools are needed to facilitate valid measurement of CIM and heightened understanding of its potential value to the field.

## Supporting information

S1 Checklist Preferred Reporting Items for Systematic reviews and Meta-Analyses extension for Scoping Reviews (PRISMA-ScR) checklist. (DOCX)

S1 Appendix Search strategy for OVID, PsycInfo, and EMBASE. (DOCX)

S1 File Included & excluded study tables. (DOCX)

S2 File Study data. (DOCX)

## Abbreviations

APA: American Psychological Association
CIM: Creativity in Medicine
PRISMA-ScR: Preferred Reporting Items for Systematic reviews and Meta-Analyses extension for Scoping Reviews
TTCT: Torrance Test of Creative Thinking
