# Supplementary material for: Creativity research in medicine and nursing: A scoping review
Source: PLoS One. 2025 Jan 8;20(1):e0317209. doi: 10.1371/journal.pone.0317209 (PMC11709234; doi:10.1371/journal.pone.0317209)
Supplement: S1 Appendix — (DOCX) [file pone.0317209.s002.docx]

**Appendices**

*S1 Appendix: Search Strategy for OVID, PsycInfo, and EMBASE*

OVID Medline Epub Ahead of Print, In-Process & Other Non-Indexed Citations, Ovid MEDLINE(R) Daily and Ovid MEDLINE(R) 1946 to 27 September 2023

1 Creativity/ 7824

2 (divergent think* or convergent think*).ti,ab. 658

3 creativ*.ti. 7066

4 1 or 2 or 3 11476

5 (medicine or medical or surgeon or doctor or physician or nurs* or clinician).ti. 918541

6 exp health personnel/ 617333

7 exp evidence-based medicine/ or exp evidence-based nursing/ or exp medicine/ or exp nursing/ or exp psychology, medical/ 1503138

8 5 or 6 or 7 2508291

9 4 and 8 **2324**

APA PsycInfo <1806 to September Week 3 2023>

1 exp creativity/ or exp creativity measurement/ 29531

2 inductive deductive reasoning/ or exp divergent thinking/ 4891

3 (divergent think* or convergent think*).ti,ab. 2149

4 creativ*.ti. 21724

5 1 or 2 or 3 or 4 37213

6 exp medical sciences/ or exp medical students/ or exp "medical treatment (general)"/ 483019

7 (medicine or medical or surgeon or doctor or nurs* or physician or clinician).ti. 107086

8 6 or 7 547723

9 5 and 8 **920**

Embase <1974 to 2023 September 26>

1 creativity/ 11139

2 (creativity or creative or divergent thinking or convergent thinking).ti. 6935

3 1 or 2 13873

4 medicine/ 46357

5 (medicine or medical or surgeon or doctor or nurs* or physician or clinician).ti. 952095

6 4 or 5 980895

7 3 and 6 **1172**
