# Supplementary material for: Creativity research in medicine and nursing: A scoping review
Source: PLoS One. 2025 Jan 8;20(1):e0317209. doi: 10.1371/journal.pone.0317209 (PMC11709234; doi:10.1371/journal.pone.0317209)
Supplement: S1 File — (DOCX) [file pone.0317209.s003.docx]

**S2 File: Included Studies**

| **Study #** | **1st Author** | **Year** | **Title** |
| --- | --- | --- | --- |
| 1 | Owen | 1970 | Achievement prediction in nursing education with cognitive, attitudinal, and divergent thinking variables. |
| 2 | Eisenman | 1970 | Creativity change in student nurses: A cross-sectional and longitudinal study. |
| 3 | Bailey | 1970 | Evaluation of the development of creative behavior in an experimental nursing program. |
| 4 | Eisenman | 1972 | Creativity in student nurses and their attitudes toward mental illness and physical disability. |
| 5 | Morrison | 1974 | Satisfaction and creative inclination in a group of British general practitioners. |
| 6 | Gough | 1976 | What happens to creative medical students?. |
| 7 | Ventura | 1976 | Creative thinking abilities of student nurses. |
| 8 | Ventura | 1979 | Use of Torrance Test of Creative Thinking to measure differences between nursing students. |
| 9 | Thomas | 1979 | Promoting creativity in nursing education. |
| 10 | Kissinger | 1981 | Nursing process, student attributes, and teaching methodologies. |
| 11 | Stephenson | 1983 | Creativity in management in family medicine. |
| 12 | Davis | 1987 | Creativity in neurosurgical publications. |
| 13 | Pesut | 1988 | Self-perceived creativity of practicing registered nurses. |
| 14 | Davis | 1990 | Creative thought in neurosurgical research: the value of citation analysis. |
| 15 | Lunney | 1992 | Divergent productive thinking factors and accuracy of nursing diagnoses. |
| 16 | Oliver | 1993 | Coloring outside the lines. |
| 17 | Berg | 1994 | Nurses' creativity, tedium and burnout during 1 year of clinical supervision and implementation of individually planned nursing care: comparisons between a ward for severely demented patients and a similar control ward. |
| 18 | Gendrop | 1996 | Effect of an intervention in synectics on the creative thinking of nurses. |
| 19 | Berg | 1999 | Effects of systematic clinical supervision on psychiatric nurses' sense of coherence, creativity, work-related strain, job satisfaction and view of the effects from clinical supervision: a pre-post test design. |
| 20 | Rothenberg | 2002 | Academic achievement and creative thinking capacity in South African medical students - An empiricial study |
| 21 | Ku | 2002 | The effectiveness of teaching strategies for creativity in a nursing concepts teaching protocol on the creative thinking of two-year RN-BSN students. |
| 22 | Kalischuk | 2002 | Thinking creatively: from nursing education to practice. |
| 23 | Jokari | 2012 | Study the effect of creativity and innovation on nurses' productivity in Taleghani hospital (case study in Abadan of Iran). |
| 24 | Tsai | 2013 | The relationship of individual characteristics, perceived worksite support and perceived creativity to clinical nurses' innovative outcome. |
| 25 | Almansa | 2013 | Thinking styles and creativity preferences in nursing. |
| 26 | Chan | 2013 | Critical thinking and creativity in nursing: learners' perspectives. |
| 27 | Lukersmith | 2013 | The perceived importance and the presence of creative potential in the health professional's work environment. |
| 28 | Kim | 2015 | Leadership, Knowledge Sharing, and Creativity: The Key Factors in Nurses' Innovative Behaviors. |
| 29 | Isfahani | 2015 | Nurses' creativity: Advantage or disadvantage |
| 30 | Isfahani | 2015 | What Really Motivates Iranian Nurses to Be Creative in Clinical Settings?: A Qualitative Study. |
| 31 | Malik | 2016 | Authentic leadership and its impact on creativity of nursing staff: A cross sectional questionnaire survey of Indian nurses and their supervisors. |
| 32 | Ku | 2016 | Develop a framework of creative thinking teaching mode for RN-BSN students on the basis of the creative process of clinical nurses in Taiwan. |
| 33 | Siri | 2017 | Ethnopsychiatry fosters creativity and the adoption of critical and reflexive thinking in higher education students: insights from a qualitative analysis of a preliminary pilot experience at the Faculty of Medicine and Surgery, University of Genoa, Italy. |
| 34 | Toyama | 2017 | Associations of trait emotional intelligence with social support, work engagement, and creativity in Japanese eldercare nurses. |
| 35 | Afsar | 2017 | Transformational leadership, creative self-efficacy, trust in supervisor, uncertainty avoidance, and innovative work behavior of nurses. |
| 36 | Tehranineshat | 2018 | The relationship between knowledge management and creativity in bachelor degree compared to master degree nursing students. |
| 37 | Park | 2018 | Quasi-experimental study on the effectiveness of a flipped classroom for teaching adult health nursing. |
| 38 | Gu | 2019 | Applying the havruta learning method to nursing education |
| 39 | Liu | 2019 | Perceived Self-Efficacy of Teaching for Creativity Among Nurse Faculty in Taiwan: A Preliminary Study. |
| 40 | Li | 2019 | Relationships among psychological capital, creative tendency, and job burnout among Chinese nurses. |
| 41 | Yang | 2019 | Effectiveness of a training program based on maker education for baccalaureate nursing students: A quasi-experimental study |
| 42 | Liu | 2019 | Nurturing and enhancing creativity of nursing students in Taiwan: A quasi-experimental study. |
| 43 | Amiri | 2020 | Creativity and its determinants among medical students. |
| 44 | Liu | 2020 | Factors affecting nursing students' creativity in Taiwan: Exploring the moderating role of creative personality |
| 45 | Liu | 2020 | Inter-professional nursing education and the roles of swift trust, interaction behaviors, and creativity: A cross-sectional questionnaire survey |
| 46 | Molero-Jurado | 2020 | Personality and job creativity in relation to engagement in nursing. |
| 47 | Liu | 2020 | Predictors of self-perceived levels of creative teaching behaviors among nursing school faculty in Taiwan: A preliminary study. |
| 48 | Liu | 2020 | The association between creativity, creative components of personality, and innovation among Taiwanese nursing students. |
| 49 | Drafahl | 2020 | The Influences Burnout and Lack of Empowerment Have on Creativity in Nursing Faculty. |
| 50 | Liu | 2020 | Effect of creativity training on teaching for creativity for nursing faculty in Taiwan: A quasi-experimental study. |
| 51 | Eaton | 2020 | Certified nursing assistants as agents of creative caregiving in long-term care. |
| 52 | Slatten | 2020 | The impact of individual creativity, psychological capital, and leadership autonomy support on hospital employees' innovative behaviour. |
| 53 | Shirazi | 2020 | Technical Simulation Using Goldfish Bowl Method: A Medical Teaching Method for Increasing Student's Creativity. |
| 54 | Barroso Alonso | 2020 | The Relationship Between Burnout and Health Professionals' Creativity, Method, and Organization. |
| 55 | Suciu | 2021 | Medical students' personalities: A critical factor for doctor-patient communication |
| 56 | Zairi | 2021 | Serious Game Design with medical students as a Learning Activity for Developing the 4Cs Skills: Communication, Collaboration, Creativity and Critical Thinking: A qualitative research |
| 57 | Ghazzawi | 2021 | Job crafting mediates the relation between creativity, personality, job autonomy and well-being in Lebanese nurses. |
| 58 | Liu | 2021 | Predictors of individually perceived levels of team creativity for teams of nursing students in Taiwan: A cross-sectional study. |
| 59 | Kim | 2021 | Social-emotional competence and academic achievement of nursing students: A canonical correlation analysis |
| 60 | Boonyoung | 2021 | Comparison of Modified Hybrid Brainstorming With a Conventional Brainstorming Program to Enhance Nurses' Innovative Idea Generation. |
| 61 | Liu | 2021 | Effect of interdisciplinary teaching on collaborative interactions among nursing student teams in Taiwan: A quasi-experimental study |
| 62 | Liu | 2021 | A mixed method evaluation of an integrated course in improving critical thinking and creative self-efficacy among nursing students. |
| 63 | Ten Haven | 2022 | Creativity: A viable and valuable competency in medicine? A qualitative exploratory study. |
| 64 | Liu | 2022 | Development and Psychometric Testing of a Taiwanese Team Interactions and Team Creativity Instrument (TITC-T) for Nursing Students. |
| 65 | Liu | 2022 | Investigating the pathways between swift trust and team creativity among nursing student teams in Taiwan: A moderated mediation model. |
| 66 | Liu | 2022 | Moderating effects of task interdependence on interaction behaviours and creativity for nursing students on interdisciplinary teams |
| 67 | Karatepe | 2022 | Nurse performance: A path model of clinical leadership, creative team climate and structural empowerment. |
| 68 | Leyva-Moral | 2022 | Nursing students' perceptions of the efficacy of narrative photography as a learning method: A cross-sectional study. |
| 69 | Liu | 2022 | The Moderating Role of Team Conflict on Teams of Nursing Students |
| 70 | Alil | 2022 | Effect of Critical Thinking Skills on Improving Creativity of Nursing Students at Technical Institute of Nursing |
| 71 | Liu | 2022 | Effectiveness of Interdisciplinary Teaching on Creativity: A Quasi-Experimental Study. |
| 72 | Liu | 2022 | Promoting creativity of nursing students in different teaching and learning settings: A quasi-experimental study. |
| 73 | Tasdelen Bas | 2022 | The effect of decorative arts course on nursing students' creativity and critical thinking dispositions. |
| 74 | Jiang | 2023 | Comparison of Trait Creativity Between Medical Students and Humanities Students. |
| 75 | Shawwa | 2023 | Does Gender, Academic Status, Years of Teaching Experience, and Discipline Affiliation Affect Strategies Used to Promote Creativity in Medical Education at King Abdulaziz University?. |
| 76 | Naveed | 2023 | How information literacy influences creative skills among medical students? The mediating role of lifelong learning. |
| 77 | Liu | 2023 | Scientific creativity and innovation ability and its determinants among medical postgraduate students in Fujian province of China: a cross sectional study. |
| 78 | Ghasemi | 2023 | Examining Coping Styles Of Nursing Students In Front Of Problems And Solving The Problem Of Depression And Other Mental Disorders |
| 79 | Xiang | 2023 | Relationship among clinical practice environment, creative self-efficacy, achievement motivation, and innovative behavior in nursing students: A cross-sectional study. |
| 80 | Toscano | 2023 | The Role of Emotional Regulation in the Relationship between Nurses' Creative Style and Innovation Behaviors: A Cross-Sectional Study. |
| 81 | Li | 2023 | Trait creativity among midwifery students: a cross-sectional study. |

**Excluded Studies During Full-Text Review (n=63; 56 studies + 7 duplicates)**

| **Study** | **Title** | **Published Year** | **Notes** |
| --- | --- | --- | --- |
| Bellosta-Batalla 2021 | Introducing mindfulness and compassion-based interventions to improve verbal creativity in students of clinical and health psychology | 2021 | Exclusion reason: Wrong population; |
| Lin 2017 | Facing the challenges in ophthalmology clerkship teaching: Is flipped classroom the answer? | 2017 | Exclusion reason: Wrong outcomes; |
| Balon 2012 | Richness and creativity in medical student education in psychiatry | 2012 | Exclusion reason: Wrong study design; |
| Anonymous 1987 | The scientific and technical creativity of young military physicians (2) | 1987 | Exclusion reason: Not published in English; |
| Kopel 2021 | Teaching methods fostering enjoyment and creativity in medical education. | 2021 | Exclusion reason: Wrong study design; |
| Fu 2022 | An Inclusive Leadership Framework to Foster Employee Creativity in the Healthcare Sector: The Role of Psychological Safety and Polychronicity. | 2022 | Exclusion reason: Wrong outcomes; |
| Gao 2022 | Effectiveness of a nursing innovation workshop at enhancing nurses' innovation abilities: A quasi-experimental study. | 2022 | Exclusion reason: Wrong construct -- Innovation; |
| Jean-Baptiste 2022 | Rethinking the Order of the Learning Process: A New and Sustainable Path Designed for an RN-to-BSN Education Program. | 2022 | Exclusion reason: Wrong study design; |
| Vykhrushch 2021 | DEVELOPMENT OF MEDICAL STUDENTS CREATIVITY AS A PRIORITY OF MODERN HIGHER EDUCATION. | 2021 | Exclusion reason: Wrong outcomes; |
| Oven 2019 | Occupational Therapists' Creativity: Tapping Into Client Centeredness Using a Novel Creativity Questionnaire. | 2019 | Exclusion reason: Wrong population; |
| Derakhshanrad 2019 | The Relationships between Problem-Solving, Creativity, and Job Burnout in Iranian Occupational Therapists. | 2019 | Exclusion reason: Wrong population; |
| Polster 2017 | An Exploratory Descriptive Study of Registered Nurse Innovation: Implications for Levels of Adoption. | 2017 | Exclusion reason: Wrong construct -- Innovation; |
| Sydorchuk 2016 | Aspects of development of leader creative thinking of medical student at the undergraduate level of medical education. | 2016 | Exclusion reason: Wrong study design; |
| Liou 2016 | Playing in the "Gutter": Cultivating Creativity in Medical Education and Practice. | 2016 | Exclusion reason: Wrong study design; |
| Raeis 2013 | Relationship between information literacy and creativity: a study of students at the isfahan university of medical sciences. | 2013 | Exclusion reason: Wrong population; |
| Shaukat 2007 | Teaching strategies and academic performances of undergraduates in quaid-I-azam medical college, bahawalpur. | 2007 | Exclusion reason: Full text unavailable; |
| Fasnacht 2003 | Creativity: a refinement of the concept for nursing practice. | 2003 | Exclusion reason: Wrong study design; |
| Lippell 2002 | Creativity and medical education. | 2002 | Exclusion reason: Wrong study design; |
| Bartley 1997 | Creativity and medicine: An atelier in medical school. | 1997 | Exclusion reason: Full text unavailable; |
| Barath 1982 | Personality correlates of creativity in Yugoslav medical students. | 1982 | Exclusion reason: Full text unavailable; |
| Barath 1980 | Structure and changes of creativity in medical students during the study. | 1980 | Exclusion reason: Full text unavailable; |
| Kirk 2004 | Nursing through the genetics lens: convergent thinking on education and professional development. | 2004 | Exclusion reason: Wrong study design; |
| Hajar 2023 | Creativity in Medicine. | 2023 | Exclusion reason: Full text unavailable; |
| Martinez 2010 | Is there a relationship between creativity and stylistic performance in a group of nursing students? | 2010 | Exclusion reason: Not published in English |
| Davis 1996 | The lived experience of creativity in nursing practice. | 1996 | Exclusion reason: Not peer reviewed; |
| Murphy 2000 | Relationship between creativity, tolerance of ambiguity, and critical thinking among undergraduate nursing students. | 2000 | Exclusion reason: Not peer reviewed; |
| Gelb 1995 | Cognitive style of creativity and organizational commitment in registered nurses. | 1995 | Exclusion reason: Not peer reviewed; |
| Sullivan 1982 | The relationship between nursing education and the creativity of nursing students: Implications for nursing education. | 1982 | Exclusion reason: Not peer reviewed; |
| Liu 2023 | Design thinking competence as self-perceived by nursing students in Taiwan: A cross-sectional study. | 2023 | Exclusion reason: Wrong outcomes; |
| Carroll 2022 | Valuing Originality in Nursing Practice. | 2022 | Exclusion reason: Wrong study design; |
| Ma 2018 | An integrative review: Developing and measuring creativity in nursing. | 2018 | Exclusion reason: Wrong study design; |
| Huang 2015 | [An experience applying the teaching strategies of cooperative learning and creative thinking in a mental-health nursing practicum for undergraduates at a technical college]. | 2015 | Exclusion reason: Not published in English; |
| Chan 2013 | A systematic review of creative thinking/creativity in nursing education. | 2013 | Exclusion reason: Wrong study design; |
| Pavill 2011 | Fostering creativity in nursing students: a blending of nursing and the arts. | 2011 | Exclusion reason: Wrong outcomes; |
| Shiomi 2009 | [Development of competency measurement concerning the creation of projects/social resources for public health nurses: investigation of reliability and validity]. | 2009 | Exclusion reason: Not published in English; |
| DeJonge 2014 | Managing employee creativity and health in nursing homes: The moderating role of matching job resources and matching occupational rewards. | 2014 | Exclusion Reason: Wrong population; |
| Miller 2008 | Cultivating creativity. | 2008 | Exclusion reason: Wrong study design; |
| Parker 2007 | Creativity and innovation: keys to quality care. | 2007 | Exclusion reason: Wrong study design; |
| Kirby 2000 | Why creativity lies at the heart of nursing care. | 2000 | Exclusion reason: Wrong study design; |
| LeStorti 1999 | Creative thinking in nursing education: preparing for tomorrow's challenges. | 1999 | Exclusion reason: Wrong study design; |
| Jacono 1996 | The benefits of Newman and Parse in helping nurse teachers determine methods to enhance student creativity. | 1996 | Exclusion reason: Wrong study design; |
| Harrison 1993 | Creativity in nurse education. | 1993 | Exclusion reason: Wrong study design; |
| Schaefer 1990 | Creativity in critical care nursing. | 1990 | Exclusion reason: Wrong study design; |
| Stepp-Gilbert 1985 | Creativity in clinical nursing. | 1985 | Exclusion reason: Wrong study design; |
| Manfredi 1981 | Why creativity in nursing?. | 1981 | Exclusion reason: Wrong study design; |
| Bowns 1971 | Promoting creativity in nursing education. | 1971 | Exclusion reason: Wrong study design |
| Alfano 1970 | Creativity in nursing practice--the challenge. | 1970 | Exclusion reason: Full text unavailable |
| Liu 2023 | Design thinking competence as self-perceived by nursing students in Taiwan: A cross-sectional study | 2023 | Exclusion reason: Wrong outcomes; |
| Shevchenko 2022 | Interrelation of lifestyle and psychological health of medical students | 2022 | Exclusion reason: Not published in English; |
| KalatehSadati 2021 | A Qualitative Study of Nursing Management in Iran | 2021 | Exclusion reason: Wrong outcomes; |
| Shariati 2020 | Inhibitory cognitive factors of creativity in medical students | 2020 | Exclusion reason: Not published in English; |
| Bang 2018 | Relationship among empathy ability, creativity confluence competency and problem-solving ability in nursing student | 2018 | Exclusion reason: Full text unavailable |
| AlbaMartin 2016 | The importance of creativity as professional competence nurse actually | 2016 | Exclusion reason: Not published in English; |
| Chan 2013 | A systematic review of creative thinking/creativity in nursing education | 2013 | Exclusion reason: Wrong study design; |
| Brabander 2012 | Expressing creativity in the nursing care units | 2012 | Exclusion reason: Full text unavailable |
| Farsky 1986 | Education to creativity in the instruction of internal medicine | 1986 | Exclusion reason: Full text unavailable; |
