# Supplementary material for: Creativity research in medicine and nursing: A scoping review
Source: PLoS One. 2025 Jan 8;20(1):e0317209. doi: 10.1371/journal.pone.0317209 (PMC11709234; doi:10.1371/journal.pone.0317209)
Supplement: S2 File — (DOCX) [file pone.0317209.s004.docx]

**S3 File:** Study Data

**Data Extractors**: Alex Thabane, Sushmitha Pallapothu, Sarah Saleh

**Date of Extraction Completed:** 29 February 2024

| **Study #** | **Country** | **Medical Specialty** | **Study Design** | **Sample Size** | **Creativity Definition (if made explicit)** | **Tool Used** |
| --- | --- | --- | --- | --- | --- | --- |
| 1 | United States | Nursing | Cohort study | 321 |  | Other: Alternate Uses Test, Creative Self-Rating Scale |
| 2 | United States | Nursing | Cohort study | 266 | The opposite of authoritarianism; a complex construct made up of several components | Other: Unusual Uses Test, Personal Opinion Survey |
| 3 | United States | Nursing | Non-randomised experimental study | 141 | Torrance's definition of creative problem solving stresses human needs. Tension is aroused by the sense of incompleteness or disharmony and a person is uncomfortable, wishing to relieve this tension. If traditional or habitual ways of behavior are inadequate, a person will begin to avoid the commonplace and obvious solutions by investigating, manipulating, or making guesses. His tension persists until these guesses or hypotheses have been tested, modified, and retested. Tension is still felt until the discovery is communicated. | Other: Torrance Tests of Creative Thinking, Torrance Common Problems Test, Torrance Social Improvements Test, Bailey General Nursing Problems Test |
| 4 | United States | Nursing | Cross sectional study | 114 | Preference for complexity in polygons |  |
| 5 | UK | Family Medicine | Cross sectional study | 40 | The creative physician was defined by the following set of characteristics: 1) he views each patient and problem as unique and adjusts his management appropriately; 2) he enjoys the moment of encounter with patients for the possibilities of thought and action which this encounter presents; 3) he is comfortable with and even enjoys ambiguity in problems; 4) he is patient and persistent in the exploration of verbal and non-verbal cues; 5) he is aware of patients emotional responses in furthering the successful resolution of problems. | Other: An original scale |
| 6 | United States | Medical Students / Medical Education | Cohort study | 284 |  | Other: Barron Welsh Art Scale, CPI Self-Acceptance Scale, CPI Good Impression Scale, CPI Achievement via Conformance Scale, SVIB Office Worker Scale, SVIB Banker scale |
| 7 | United States | Nursing | Cross sectional study | 335 |  | Torrance Test of Creative Thinking (TCTT) |
| 8 | United States | Nursing | Cross sectional study | 344 | Ability to bring into existence something new and unconventional and the process of doing so | Torrance Test of Creative Thinking (TCTT) |
| 9 | United States | Nursing | Non-randomised experimental study | 192 |  | Torrance Test of Creative Thinking (TCTT) |
| 10 | United States | Nursing | Cohort study | 201 |  | Other: Topics Test, Different Uses Test |
| 11 | Canada | Family Medicine | Cross sectional study | 16 | Creativity can be considered from many viewpoints. Rogers4 stated that â€œ the creative process is the emergence in action of a novel relaÂ­tion or product growing out of the uniqueness of the individual on one hand and the materials, events, persons and circumstances of his life on the other.â€ This definition includes three compoÂ­nents of creativity: a product, a process, and a personality characteristic. Product criteria refer to the end product of the creative process and specifÂ­ically to the attributes of the products produced in terms of their uncommonness and novelty. ProcÂ­ess criteria refer to the thinking styles involved in creativity, an example of which would be GuilÂ­fordâ€™s5 divergent thinking. Personality criteria refer to specific aspects of a personâ€™s personality that are said to make that person creative. An exÂ­ample would be a greater tolerance of uncertainty in a more creative person. This study concentrated on creativity as a personality characteristic. | Other: Barron Welsh Art Scale |
| 12 | United States | Surgery | Systematic review | 50 | For the purposes of this study, "creative" has been defined as an original or unique idea that conforms with the facts or reality either as an abstraction or application and that becomes an addition to what can be referred to as our "cultural heritage" |  |
| 13 | United States | Nursing | Cross sectional study | 28 |  | Other: What Kind of Person Are You? |
| 14 | United States | Surgery | Systematic review | 39 | The capacity for original or unique ideas that conform to the facts or reality either as an abstraction or application |  |
| 15 | United States | Nursing | Case report | 86 |  | Other: Utility Test and Possible Jobs Test |
| 16 | United States | Nursing | Qualitative research | 48 | The ability to see problems in a different way and to generate new or significant solutions for resolving problems |  |
| 17 | Other: Sweden | Nursing | Non-randomised experimental study | 39 |  | Other: Creative Climate Questionnaire (CCQ) |
| 18 | United States | Nursing | Other: Quasi-experimental study | 97 |  | Other: Torrance Test of Creative Thinking, Gordon Creative Problem Solving Test |
| 19 | Other: Sweden | Nursing | Other: Prepost-test design | 22 |  | Other: Creative Climate Questionnaire (CCQ) |
| 20 | Other: South Africa | Medical Students / Medical Education | Cross sectional study | 22 |  | Other: Word association test from Kent and Rosanoff |
| 21 | Taiwan | Nursing | Non-randomised experimental study | 52 | Creativity is a complex of cognitive processes, traits, skills, and capacities that include knowledge, thinking styles such as unconventional thinking, personality traits such as curiosity, and motivation such as working autonomously. Creative thinking is an intellectual ability beyond the IQ, consisting of fluency, flexibility, uniqueness, sensitivity, and elaboration (according to Guilford and the SI) | Other: Creativity in the Application of the Nursing Process Tool (CNPT) |
| 22 | Canada | Nursing | Qualitative research | 14 | "Creativity is the actualizing of our potential... It is the integration of our logical side with our intuitive side...Creativity is more than mere spontaneity for it involves deliberation as well" |  |
| 23 | Other: Iran | Nursing | Other: Case Study | 103 | A main aspect of thinking; the presentation of novel thought or plan for quliattive or quantitative improvement of organization activities; Creativity is presentation of novel plans for new peoducts and services and sustainability after the absence of those phenomenons; Creativity means the ability to present a new solution to solve problems |  |
| 24 | Taiwan | Nursing | Cross sectional study | 538 | Creativity can be seen as an innate ability, whereas innovation is the interactive result of perceived creativity and the environment; in other words, an innovative person has the ability not only to generate new ideas but also to transform the ideas into new products or services | Other: 8-item scale by Lin 2007 |
| 25 | Other: Spain | Nursing | Cross sectional study | 229 |  | Other: CREA (spanish test of creative intelligence) |
| 26 | Other: Hong Kong | Nursing | Qualitative research | 36 | The definition of creativity is very broad and Lau (2011) summarised the meaning of creativity by three principles: (i) new rearrangement of old ideas; (ii) selection of useful ideas; and (iii) exploration of connections among ideas. |  |
| 27 | Australia | Other: 6% occupational therapists, 16% speech pathologists and 11%physiotherapists. The remaining 27% included social workers, psychologists, registered nurses, exercise physiologists, dieticians, doctors, podiatrists, health promotion officers, prosthetists, residential or care worker | Cross sectional study | 361 | Creativity refers to the development of novel, potentially useful ideas | Other: Adapted from the Creativity Development Quick Scan Instrument (CDQS) |
| 28 | Other: South Korea | Nursing | Cross sectional study | 347 | Creative self-efficacy is defined as confidence in one's ability and includes creative problems solving based on the situation | Other: 8-item Questionnaire by Carmeli and Schaubroeck |
| 29 | Other: Iran | Nursing | Qualitative research | 14 | Creativity is the generation of novel and useful ideas |  |
| 30 | Other: Iran | Nursing | Qualitative research | 16 | Creativity is defined as the production of novel and useful ideas in any domain. Creative ideas are starting points of all innovations. Baron and Tang (2011) also argue that creativity is a necessary condition for subsequent innovations. Creativity is a function of three components: expertise, creative-thinking skills, and motivation |  |
| 31 | Other: India | Nursing | Cross sectional study | 486 | Creativity may be understood as creation of new usable knowledge or effective and novel use of existing knowledge | Other: 3-item scale by Oldham and Cummings |
| 32 | Taiwan | Nursing | Qualitative research | 18 | Ability to develop an innovation on the basis of old knowledge. process of understanding problems, producing ideas and designing activities by applying divergent and convergent thinking. Chao, Tai, and Chiu(2005) defined creativity as inspiring fluency, flexibility and uniqueness and applying the knowledge to associate, transform or reconstitute to fit the current needs or functional behaviours. |  |
| 33 | Other: Italy | Medical Students / Medical Education | Randomised controlled trial | 30 | Creativity, generally meant as ability to produce something new or as a way to interpret in a new and original way, recasting, and reframing what has been already realized or acquired. |  |
| 34 | Other: Japan | Nursing | Cross sectional study | 489 |  | Other: Bew Brief Job Stress Creativity |
| 35 | Other: Pakistan | Nursing | Cross sectional study | 1064 |  | Other: Creative self-efficacy scale by Tierney and Farmer |
| 36 | Other: Iran | Nursing | Cross sectional study | 180 |  | Other: Randsips Creativity Questionnaires |
| 37 | Other: South Korea | Nursing | Non-randomised experimental study | 81 |  | Other: Critical Thinking Disposition Scale |
| 38 | Other: South Korea | Nursing | Cross sectional study | 146 | Creativity is one of the goals in education to prepare students for the future, and it is stressed as a key competency that learners should develop. Nurses are also required to develop problem-solving abilities through creative thinking, and creativity in educational and work-related work is considered an important job competency for nurses |  |
| 39 | Taiwan | Nursing | Cross sectional study | 53 | Creativity, the process of developing something new and valuable. | Other: Affective Components of Creativity Scale (ACCS), Creativity Teaching Efficiency of Technology Institute Teacher's Scale (CTETITS), Self-Efficacy for Creativity Teaching Scale (SECTS) |
| 40 | Other: China | Nursing | Cross sectional study | 200 | Creative tendency as a positive psychological tendency or creative personality towards creativity (Shen, Wang, & Shi, 2005), is regarded as a necessary component of creativity | Other: Creativity Assessment Packet |
| 41 | Other: China | Nursing | Non-randomised experimental study | 40 |  | Other: Williams Creative Scale |
| 42 | Taiwan | Nursing | Non-randomised experimental study | 245 | Creativity is the process of developing something new and valuable | Other: TTCT; Affective Components of Creativity Scale |
| 43 | Other: Iran | Medical Students / Medical Education | Cross sectional study | 720 | The most important attributes mentioned for creative people are the fluency of thought, flexibility, innovation, the ability to create new definitions of problems, and sensitivity to problems, and the creative people are people whose new ideas are recognized and accepted by others. | Guilford Creativity Questionnaire |
| 44 | Taiwan | Nursing | Cross sectional study | 319 | has to do with creative thinking and creative personality traits, not clearly defined. | Other: School Creative Climate Scale, Torrance Test of Creative Thinking, Creative Personality Scale |
| 45 | Taiwan | Nursing | Cross sectional study | 210 |  | Other: Farh and Yang team creativity scale |
| 46 | Other: Spain | Nursing | Cross sectional study | 1268 |  | Other: Creative Environment Perceptions Scale (CEP) |
| 47 | Taiwan | Nursing | Cross sectional study | 53 | Creative teaching behaviors can be defined as those acts that fosterstudent creativity. These behaviors include those that stimulate independent learning, cooperation, and provide a solid base for divergent thinking; encourage students to take time to formulate ideas and think flexibly; promote self-evaluation, listening, and questioning; and help students learn to cope with frustration and failure | Other: Creative Teaching Behaviour Scale |
| 48 | Taiwan | Nursing | Cross sectional study | 98 |  | Other: Torrance Test of Creative Thinking, Affective Components of Creativity Scale, Consensual Assessment Technique |
| 49 | United States | Nursing | Cross sectional study | 210 | The literature search identified a limited number of studies that defined creativity, but few that examined creativity and nursing. Creativity was used as an adjective in several articles that mentioned critical thinkers stated that, although people possess some form of creativity, cognitive creativity stems from a cognitive or thinking process that seeks to create innovative new concepts. This process requires the individual to have the knowledge, intellectual ability, personality, insight ability, and motivation to think in a new way. | Other: Gough Creative Personality Scale (CPS) |
| 50 | Taiwan | Nursing | Non-randomised experimental study | 42 |  | Other: Creativity Teaching Behavior Scale (CTBS); Creativity Teaching Efficiency of Technology Institute Teacher's Scale(CTETITS); Self-efficacy for Creativity Teaching Scale (SECTS) |
| 51 | United States | Nursing | Qualitative research | 14 | Certified nursing assistants initially defined creativity as a formal experience led by activity directors and requiring setâ€up, â€œ...they [CNAs] just have so much to do that they're not necessarily taking the time to set up that type of thing.â€ Some felt that creativity was only practiced by those with natural abilities. â€œSo I think Iâ€™ve seen it at times where it's quite prevalent. And Iâ€™ve seen it at times where's it's not. And I think that's truly staff based on who's got what ability and how to do it.â€ Others felt that learning creativity is possible, and often gained through life experience, maturity, motivation and using common sense. Certified nursing assistants felt that nurses should consult them more for creative insight on approaching residents. â€œFor me, I think it would eliminate sometimes medicating them, if they would come and ask the CNA how we're approaching them.â€ |  |
| 52 | Other: Norway | Other: Doctors, Nurses, Other (Admin staff, other health professionals) | Cross sectional study | 1008 | Creativity is most often described as a necessary input to innovation. SlÃ¥tten and Mehmetoglu, emphasizing the importance of creativity, characterized it as a primary source [22] of innovative behaviour. Gilmartin illustrates the criticality of creativity by describing it as the fuel of innovation [23]. The foundation of innovation ideas creativity | Other: Items adapted from Zhou and George |
| 53 | Other: Iran | Other: Midwifery | Non-randomised experimental study | 70 | Creativity is multi-dimensional and potential. This is why it cannot be measured easily with a tool and requires the use of different techniques and comprehensive tools.12Creativity has three components, including intrinsic motivation, skills associated with relevant expertise and cognitive processes | Dr. Abedi's Standardized Creative Thinking Questionnaire |
| 54 | Other: Spain | Surgery | Cross sectional study | 70 |  | Other: CREA |
| 55 | Other: Romania | Medical Students / Medical Education | Cross sectional study | 119 |  | Other: Barron Welsh Art Scale |
| 56 | Other: Tunisia | Medical Students / Medical Education | Qualitative research | 12 |  |  |
| 57 | Other: Lebanon | Nursing | Cross sectional study | 547 | Creativity has been frequently used to define JC, as the latter is a set of creative actions demonstrated by employees at work | Other: 5-point Likert Scale |
| 58 | Taiwan | Nursing | Cross sectional study | 99 | Team creativity may be defined as stemming from individual team members' creativity or as a culmination of complex interactions among the group as a whole. | Other: TCTT; and Team Creativity instrument developed by Farh and adapted by Yang for Chinese populations |
| 59 | Other: South Korea | Nursing | Cross sectional study | 195 |  | Other: Runco Ideational Behaviour Scale |
| 60 | Other: Thailand | Nursing | Non-randomised experimental study | 56 |  | Other: Measured by 'Innovation Experts' for fluency, flexibility and originality |
| 61 | Taiwan | Nursing | Non-randomised experimental study | 145 |  |  |
| 62 | Other: China | Nursing | Other: Mixed-methods | 68 | "Creative thinking is an attribute influencing personal and societal prospects (Huang et al., 2020). Creative self-efficacy is an essential component of creative thinking, which refers to individuals' beliefs about their ability to generate novel and adaptive ideas, solutions, and produce creative things (Tierney and Farmer, 2002)." | Other: Creative Self-Efficacy Scale |
| 63 | Other: Netherlands | Medical Students / Medical Education | Qualitative research | 34 | As a term widely used, creativity is generally considered to be multifaceted, although its definition differs depending on the context ( |  |
| 64 | Taiwan | Nursing | Cross sectional study | 275 |  | Other: Taiwanese Team Interactions and Team Creativity Scale (TITC-T) |
| 65 | Taiwan | Nursing | Cross sectional study | 629 |  | Other: Team Creativity Scale by Farth |
| 66 | Taiwan | Nursing | Cross sectional study | 99 |  | Other: Farh and Li team creativity scale |
| 67 | Other: Turkey | Nursing | Cross sectional study | 664 | Creativity is the ability to discover new perspectives on problems and opportunities, develop new ideas, create solutions to problems and implement these solutions | Other: Creative Team Climate Scale (CTCS) |
| 68 | Other: Spain | Nursing | Cross sectional study | 66 | The present results indicate that NP is a learning method that elicits a high degree of satisfaction among students, an outcome that was also reported in previous works (Leyva-Moral et al., 2019; Parker, 2021). This study also found that NP helps students assimilate existing theoretical concepts and also promotes their creativity |  |
| 69 | Taiwan | Nursing | Cross sectional study | 270 |  | Other: Farh and Li team creativity scale |
| 70 | Other: Egypt | Nursing | Non-randomised experimental study | 267 |  | Other: Kirton Adaption-Innovation Inventory |
| 71 | Taiwan | Nursing | Non-randomised experimental study | 145 |  | Other: TCTT and 10-item team creativity scale developed by Yang, based on Farh |
| 72 | Taiwan | Nursing | Non-randomised experimental study | 191 |  | Torrance Test of Creative Thinking (TCTT) |
| 73 | Other: Turkey | Nursing | Non-randomised experimental study | 141 |  | Other: Marmara Creative Thinking Dispositions Scale |
| 74 | Other: China | Medical Students / Medical Education | Cross sectional study | 701 |  | Other: Williams Creativity Assessment Packet |
| 75 | Other: Saudi Arabia | Medical Students / Medical Education | Cross sectional study | 213 |  |  |
| 76 | Other: Pakistan | Medical Students / Medical Education | Cross sectional study | 473 | "the production of new, original, and valuable ideas" | Other: Creativity scale by Munoz-Doyague et al |
| 77 | Other: China | Medical Students / Medical Education | Cross sectional study | 1241 |  | Other: Developed by researchers themselves |
| 78 | Other: Iran | Nursing | Cohort study | 118 |  | Other: Cassidy and Long Problem Solving Scale |
| 79 | Other: China | Nursing | Cross sectional study | 588 |  | Other: Creative Self-Efficacy Scale |
| 80 | Other: Iran | Nursing | Cross sectional study | 187 |  | Other: Problem-Solving Style Questionnaire adapted by Cassidy and Burnside |
| 81 | Other: China | Other: Midwifery | Cross sectional study | 300 | "a person's ability to generate an idea or a product that is deemed as both novel and appropriate by experts in a field of human activities." | Other: Williams Creativity Assessment Packet |
